# Temperature and species richness effects in phytoplankton communities

**DOI:** 10.1007/s00442-012-2419-4

**Published:** 2012-08-01

**Authors:** Stefanie Schabhüttl, Peter Hingsamer, Gabriele Weigelhofer, Thomas Hein, Achim Weigert, Maren Striebel

**Affiliations:** 1 WasserCluster Lunz, Dr. Carl Kupelwieser Promenade 5, 3293 Lunz am See, Austria; 2 Department of Limnology, University of Vienna, Althanstrasse 14, 1090 Vienna, Austria; 3 Department of Organismic Biology, University of Salzburg, Hellbrunnerstrasse 34, 5020 Salzburg, Austria; 4 Institute of Hydrobiology and Aquatic Ecosystem Management, University of Natural Resources and Life Sciences, Max Emanuel-Strasse 17, 1180 Vienna, Austria; 5 Department Biology II, Ludwig-Maximilians University Munich, Grosshaderner Strasse 2, 82152 Planegg-Martinsried, Germany

**Keywords:** Biodiversity, Overyielding, Complementarity effect, Diversity–stability, Selection effect

## Abstract

Phytoplankton play an important role as primary producers and thus can affect higher trophic levels. Phytoplankton growth and diversity may, besides other factors, be controlled by seasonal temperature changes and increasing water temperatures. In this study, we investigated the combined effects of temperature and diversity on phytoplankton growth. In a controlled laboratory experiment, monocultures of 15 freshwater phytoplankton taxa (green algae, cyanobacteria, and diatoms) as well as 25 mixed communities of different species richness (2–12 species) and taxa composition were exposed to constant temperatures of 12, 18, and 24 °C. Additionally, they were exposed to short-term daily temperature peaks of +4 °C. Increased species richness had a positive effect on phytoplankton growth rates and phosphorous content at all temperature levels, with maximum values occurring at 18 °C. Overyielding was observed at almost all temperature levels and could mostly be explained by complementary traits. Higher temperatures resulted in higher fractions of cyanobacteria in communities. This negative effect of temperature on phytoplankton diversity following a shift in community composition was most obvious in communities adapted to cooler temperatures, pointing to the assumption that relative temperature changes may be more important than absolute ones.

**Electronic supplementary material:**

The online version of this article (doi:10.1007/s00442-012-2419-4) contains supplementary material, which is available to authorized users.

## Introduction

Biodiversity is an essential prerequisite for the maintenance of ecosystem services providing manifold benefits to human well being (Millennium Ecosystem Assessment 2005). With regard to phytoplankton biodiversity, these benefits may reach from supporting primary productivity and provisioning food (via the trophic cascade) to regulating water quality and climate (e.g., via nutrient and carbon fixation, oxygen production), as well as aesthetic and health aspects (e.g., preventing cyanobacteria dominance and blooms in water bodies used for recreational purposes). Moreover, biodiversity minimizes negative responses to environmental changes by enhancing the resistance and resilience of ecosystems (McNaughton 1977; Naeem and Li 1997; Chapin et al. 2000). However, biodiversity is decreasing at a rapid pace (Hooper et al. 2005; Worm et al. ;^2006^) and this decrease is further accelerated by man-made climate change and associated rising temperatures on a global scale (Lovejoy and Hannah 2006; IPCC 2007).

Phytoplankton patterns vary across ecosystems, have large year-to-year variability, and show annual cycles of biomass variability and recurrence strength (Scheffer 1991; Paerl and Huisman 2008; Winder and Cloern 2010). Temperature is among the major determinants to influence phytoplankton growth rates, nutrient stoichiometry, and spatial and temporal distribution in freshwater systems. In addition to factors such as nutrient availability, light, or grazing pressure, phytoplankton taxa respond differently to different temperature regimes (e.g., Canale and Vogel 1974; Butterwick et al. 2005). With global average surface temperatures projected to increase by 1.1–6.4 °C within the next 100 years (IPCC 2007), water temperatures will follow this trend, leading to alterations in the thermal regime of many freshwater habitats (Webb and Nobilis 2007) and amplify changes in both phytoplankton community structure and distribution in especially sensitive aquatic ecosystems like small and shallow water bodies or mountain lakes.

Despite representing only 0.2 % of global primary producer biomass, phytoplankton holds responsible for about half of the world’s primary production and is thus a key player in the global carbon cycle (Field et al. 1998; Geider et al. 2001). Such high productivity is supported by fast turnover rates of aquatic primary producers (usually a few days), which, however, may also account for the extreme sensitivity and immediate reaction of phytoplankton systems towards external environmental forces (Falkowski et al. 1998).

According to the diversity–stability hypothesis, taxonomic, functional, and genetic diversity exert stabilizing effects on ecosystem functions like productivity or resource use efficiency (McCann 2000). The number of studies dealing with diversity–stability relationships in aquatic systems lag behind those of terrestrial ecosystems, but ongoing research is testing whether well-studied principles for terrestrial ecosystems hold true at the base of aquatic food webs (Naeem and Li 1997; Ptacnik et al. 2008; Striebel et al. 2009b). Even though the relationship between taxa and/or functional diversity and ecosystem stability has controversially been discussed (Lehman and Tilman 2000; Pfisterer and Schmid 2002), in agreement with the diversity–stability hypothesis, algal communities of high species and functional richness have been shown to lead to increased productivity and resource use efficiency (Ptacnik et al. 2008; Striebel et al. 2009a; Cardinale 2011). This positive biodiversity effect (overyielding) means that the yield of a mixed community turns out higher than expected based on the average yield of the species contained in the community, or higher than the yield of any monoculture of the community’s species (Hector et al. 2002). In general, two major mechanisms are suggested to be responsible for increased productivity at high diversity: (1) complementarity and (2) selection for species with particular traits (Tilman et al. 1997; Loreau 2000; Loreau and Hector 2001). Complementarity means that resource partitioning and/or facilitation among species leads to increased resource use and productivity in more diverse communities (Loreau and Hector 2001; Cardinale et al. 2006; Tilman et al. 2006). Selection, on the other hand, occurs when certain species in a mixture become dominant due to beneficial trait combinations, and in turn constitute the bulk of the community biomass.

Most studies investigating the effect of temperature on plankton communities have focused on the effects of temperature on the mixing of the stratified water column and the resulting effects of light limitation on phytoplankton, especially in deep lakes (Diehl 2002; Berger et al. 2007; Tirok and Gaedke 2007) or on the coupling of phytoplankton and zooplankton growth (Berger et al. 2007; Sommer et al. 2007). In the light of seasonal temperature changes and changing climate conditions, direct temperature effects become more relevant, and it is of particular interest whether biodiversity, and in turn performance and resistance, can be sustained in phytoplankton communities experiencing stress conditions such as temperature increases.

In this study, we therefore investigated the combined effects of temperature and species diversity on phytoplankton growth performance, nutrient dynamics, and community composition based on the following hypotheses:

1. Following the diversity–stability concept, growth rates and phosphorus content of phytoplankton communities increase with increasing diversity, independent of temperature.
2. Communities of higher diversity show a positive net biodiversity effect (overyielding) due to complementarity and/or selection effects.
3. Distinctive temperature-dependent growth performance of taxonomically different phytoplankton groups leads to a temperature-related shift in community composition due to selection for more heat-tolerant groups at higher temperatures.
4. As a consequence of (3), increased temperature results in a decrease in diversity.

## Materials and methods

### Experimental set-up

We performed laboratory experiments with 15 pre-cultured phytoplankton species from three taxonomic groups (Chlorophyceae (green algae), Cyanophyceae (cyanobacteria), Bacillariophyceae (diatoms)) (Online Resource Table S1). Cultures were obtained from various algal culture collections and grown in WC growth medium (Guillard and Lorenzen 1972). According to a naturally occurring temperature range, three basic temperature levels were established in a climate chamber: 12, 18, and 24 °C. Cell culture flasks (250 mL) were used as experimental units, shaken twice a day to reduce sinking losses, and positions of culture flasks were permuted randomly to guarantee equal conditions. The semi-batch cultures (medium exchange of 10 % day^−1^) were exposed to a light:dark cycle of 16:8 h at a light intensity of about 100 μmol m^−2 ^s^−1^ (measured in water). Light intensity and water temperature were controlled through continuous data logging.

### Experimental phase I—incubation at constant temperatures (12, 18, 24 °C)

In phase I of the experiment, 200 mL of each of the 15 monocultures (with three replicates each) were transferred to the cell culture flasks at equal initial biovolume (3 × 10^6^ μm^3^ mL^−1^, a value comparable to natural phytoplankton concentrations) and grown for 14 days to adapt to constant 12, 18, and 24 °C, respectively.

Additionally, we established artificial mixed communities of different species richness at five levels (2, 3, 6, 9, and 12 species), each with five randomly assigned species combinations based on our species pool (see Online Resource Table S2) resulting in a total of 25 mixed communities. We started the experiment at equal total chlorophyll content per treatment and with even partitioning of the species contained in a mixture. In order to obtain a feasible amount of samples, we reduced the number of replicates: one mixture per level was randomly chosen to be replicated three times, while the remaining four mixtures per level were not replicated (Online Resource Table S2). For each level, the coefficient of variation of both POC (particulate organic carbon) and POP (particulate organic phosphorous) within the three replicates was lower than the coefficient of variation between all five treatments per level (see Online Resource Table S3). This shows that the coefficient of variation within different species was higher than the stochastic effect. Thus, for further data analysis, five treatments per level without replicates were used.

### Experimental phase II—temperature increases

Starting on day 15 (after the cultures had reached their stationary growth phase), water temperature for both the monocultures and the communities was increased by 4 °C over a period of 7 h per day to reach 16, 22, and 28 °C, respectively. These temperature peaks were repeated daily for 7 days (experimental setup see Online Resource Fig. S1) to simulate strong daily temperature variation in sensitive (small and shallow) water bodies.

### Sampling and analysis

As proxies for biomass and nutrient uptake, respectively, samples for POC and POP analysis were filtrated onto precombusted and acid-washed glass-fiber filters (Whatman GF/C) at the start (*t*_0_), after phase I (*t*_1_), and again after phase II (*t*_2_). POC was measured by infrared spectrometry (C-Mat 5500; Ströhlein), and POP by molybdate reaction after sulfuric acid digestion (Wetzel and Likens 2003). Total phosphorus was also measured by molybdate reaction after sulfuric acid digestion. To determine cell numbers and biovolumes, community samples were taken at *t*_0_, *t*_1_, and *t*_2_, and were fixed with Lugol’s solution to be counted under an inverted microscope (Utermöhl 1958). Species-specific cell volumes were calculated by approximation to simple geometrical bodies (Hillebrand et al. 1999). Community biovolumes were calculated as the product of single cell volumes with corresponding cell densities derived from Utermöhl counting.

### Data analyses

Biomass gain was used to calculate growth rates (*r*) based on an exponential function over time, where POC was used as a proxy for biomass at the start (*t*_0_) and at the end (*t*_1_ and *t*_2,_ respectively). A one-way ANOVA was performed to test for species richness effects on growth rate and POP concentrations at *t*_1_ and *t*_2_. Saturation curves [*y* = *a* × *x*/(*b* + *x*)] were assumed between species richness (*x*) and growth rate or POP concentration (*y*), respectively, and were tested against linear regressions (*y* = *a* + *b* × *x*) using one-way ANOVA. By comparing two regressions by one-way ANOVA algorithm, the variances of residuals of regressions were compared, with regressions having the same independent (species richness) and dependent (growth rates/POP concentrations) variable. By using this method, the regression providing the significantly better fit could be found.

To separate between potential effects leading to the observed net effect, we calculated the contributions of the selection and complementarity effects based on community biovolume according to additive partitioning suggested by Loreau and Hector (2001). Biodiversity effects were plotted against species richness and, again, a hyperbolic versus a linear function was tested. Two-way ANOVAs between the factors species richness and temperature were performed for net, complementarity, and selection effects.

Monocultures were pooled according to taxonomic groups (green algae, cyanobacteria, and diatoms), and a 2-way ANOVA was performed followed by post hoc tests to find effects of temperature and taxonomic groups, as well as interactions of these two parameters at both *t*_1_ and *t*_2_.

To test for effects of temperature on the relative biovolume development in mixed communities, an algal response factor was calculated on a taxonomic group level (Sarnelle 1992). This response factor is defined as the biovolume fraction of one taxonomic group within the community at *t*_1_ and *t*_2_, respectively, divided by the group’s initial biovolume fraction at *t*_0_.

Diversity of communities based on biovolume data was calculated as the Shannon Index of diversity (*H*′). The net biodiversity effect (potential overyielding) was determined as the difference between the observed yield (based on POC concentrations and biovolume data) of the community and its expected yield calculated based on monoculture data (Loreau and Hector 2001). Statistical analyses were performed using SPSS (15.0) and R 2.13 (R Development Core Team 2008); graphs were generated in SigmaPlot (11.0).

## Results

### Growth rates and phosphorus content of phytoplankton

**Fig. 1:**
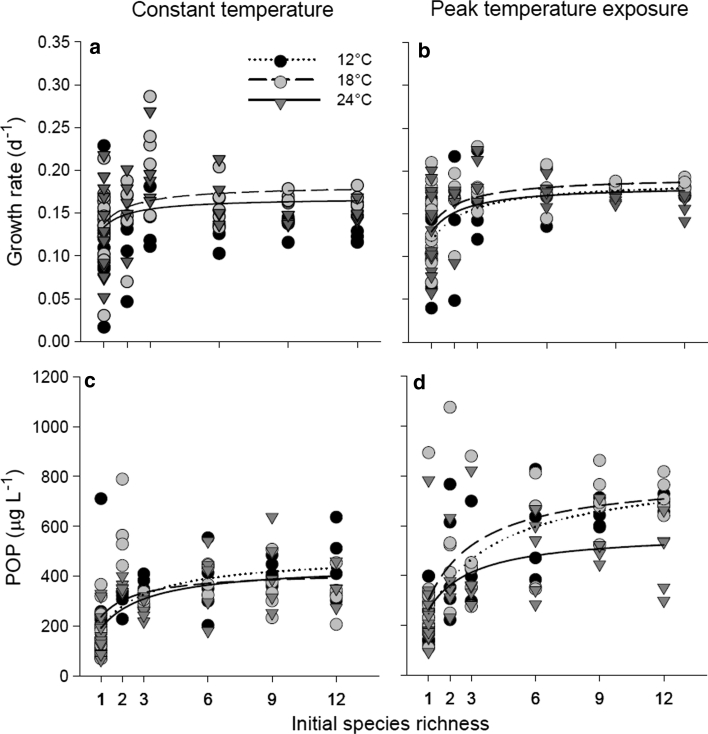
**a**, **b** Growth rates and **c**, **d** particulate organic phosphorus (POP) concentrations in response to initial species richness: **a**, **c** after 2 weeks of constant temperatures at 12, 18, or 24 °C (*t*_1_) and **b**, **d** after an additional week of short-term temperature peaks of +4 °C (*t*_2_). Significant saturation curves (*y* = *a* × *x/*(*b* + *x*) are displayed; *n* = 40; variables, see Online Resource Table S4)

Species richness had a positive effect on growth rate at all temperature levels for both, *t*_1_ and *t*_2_ best described by a saturation curve, although the effect at 12 °C at *t*_1_ was not significant (Fig. 1a, b; Online Resource Table S4). A one-way ANOVA revealed significant effects of species richness on growth rate at *t*_1_ (*P* < 0.001, *F*_5,102_ = 5.611) and *t*_2_ (*P* < 0.001, *F*_5,102_ = 11.11). A significant temperature effect could only be detected for *t*_1_ (*P* < 0.01, *F*_2,102_ = 6.18); growth rates were highest at 18 °C (Fig. 1a).

Particulate organic phosphorous concentrations significantly increased with increasing species richness at *t*_1_ and *t*_2_ and were best described by a saturation curve (Fig. 1c, d; Online Resource Table S4). A one-way ANOVA showed significant effects of species richness on POP concentrations at *t*_1_ (*P* < 0.001 *F*_5,102_ = 22.99), and *t*_2_ (*P* < 0.001 *F*_5,102_ = 20.84). Additionally, a significant effect of temperature on POP concentrations existed at *t*_2_ (*P* < 0.05, *F*_2,102_ = 3.88); POP concentrations were lowest at 24 °C.

### Mechanisms: complementarity and selection

Two-way ANOVAs with species richness and temperature as factors were performed for each net, complementarity, and selection effect. No significant effects of temperature were found, whereas species richness showed significant effects in two of six cases and one interaction was significant.

**Fig. 2:**
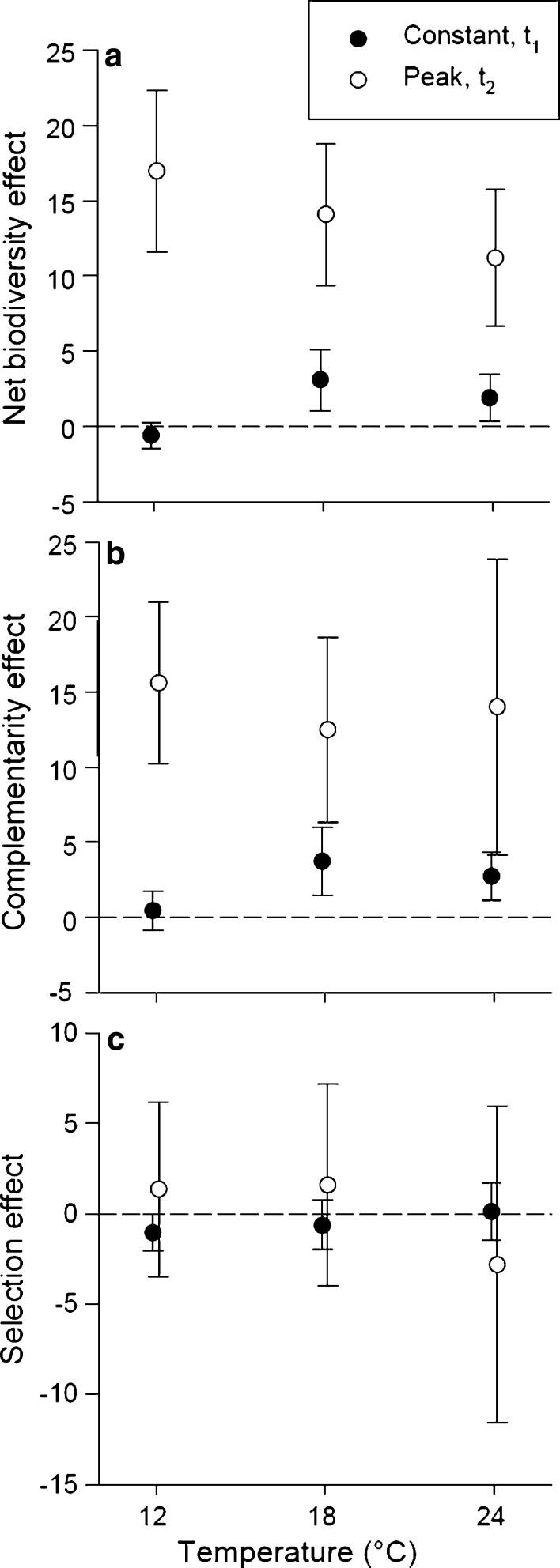
**a** Net biodiversity effect, **b** complementarity effect, and **c** selection effect of all taxa (mean ± 95 % CI) in response to constant or peak temperature exposure. Communities were exposed to 2 weeks of constant temperatures at *t*_1_ (constant, *filled dots*) and one additional week of short-term temperature peaks at *t*_2_ (peak, *open dots*). Means significantly higher than zero indicate overyielding. Symbols are slightly displaced for better illustration. Note different *y-axis* scaling for selection effect

However, these results showed no general pattern: species richness had a significant effect on complementarity at *t*_1_ (*P* = 0.025) and on selection at *t*_2_ (*P* = 0.048), and one interaction between species richness and temperature on complementarity could be revealed at *t*_2_ (*P* = 0.047; Online Resource Fig. S2). The net effect was mainly positive (Fig. 2) except at *t*_1_ 12 °C and this implies overyielding. The same pattern could be observed for the complementarity effect (positive except at *t*_1_ 12 °C; Fig. 2), whereas the selection effect was not significantly different from zero (95 % CI; Fig. 2).

### Taxonomic group-specific growth

**Fig. 3:**
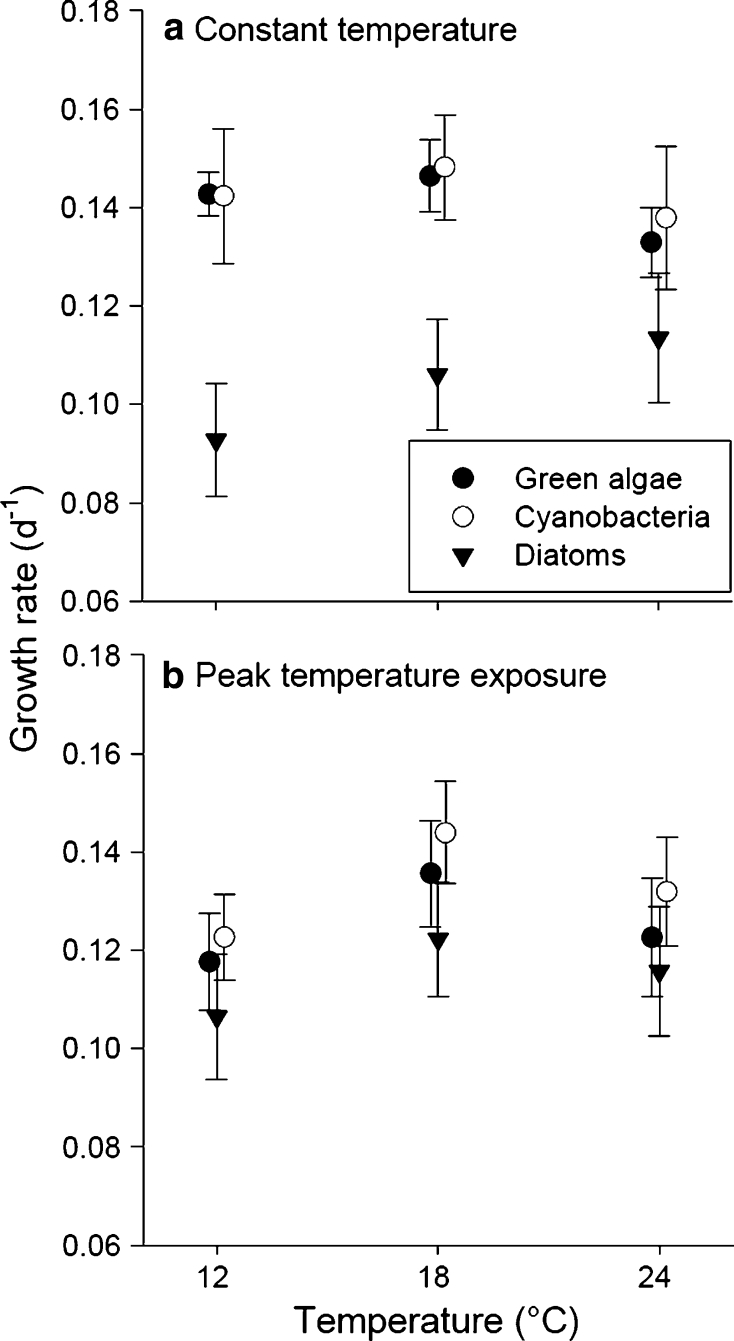
Growth rates (mean ± SE) in monocultures of green algae (*filled dots*), cyanobacteria (*open dots*), and diatoms (*triangles*) at 12, 18, and 24 °C, **a** after 2 weeks of constant temperatures at *t*_1_ and **b** after an additional week of short-term temperature peaks at *t*_2_. Symbols are slightly displaced for better illustration

After 2 weeks of incubation at constant temperatures (*t*_1_), as well as after the short-term temperature peaks (*t*_2_), monocultures showed distinguished growth rates at each of the three temperature levels (Online Resource Fig. S3). No significant effects of temperature on the growth rates after pooling all data obtained from monocultures in respect to their taxonomic groups were found at *t*_1_ and *t*_2_ (Fig. 3). However, an effect of taxonomic group (TG) was found at *t*_1_ (2-way ANOVA for *t*_1_: TG *P* < 0.001; temperature *P* = 0.58; interaction *P* = 0.44; for *t*_2_: TG *P* = 0.14; temperature *P* = 0.15; interaction *P* = 0.99; Fig. 3).

Post hoc tests for each temperature level testing for differences between taxonomic groups showed that growth rates of diatoms were significantly lower at 12 °C (post hoc test *P* < 0.01) and 18 °C (post hoc test *P* < 0.01) at *t*_1_ compared to green algae and cyanobacteria. Although not statistically significant, diatom growth rates tended to increase with increasing temperature.

### Algal response factor—relative changes in biovolume fractions in communities

The algal response factor was calculated to detect relative changes in community composition due to differences in temperature based on shifts in taxonomic group biovolume fractions.

**Fig. 4:**
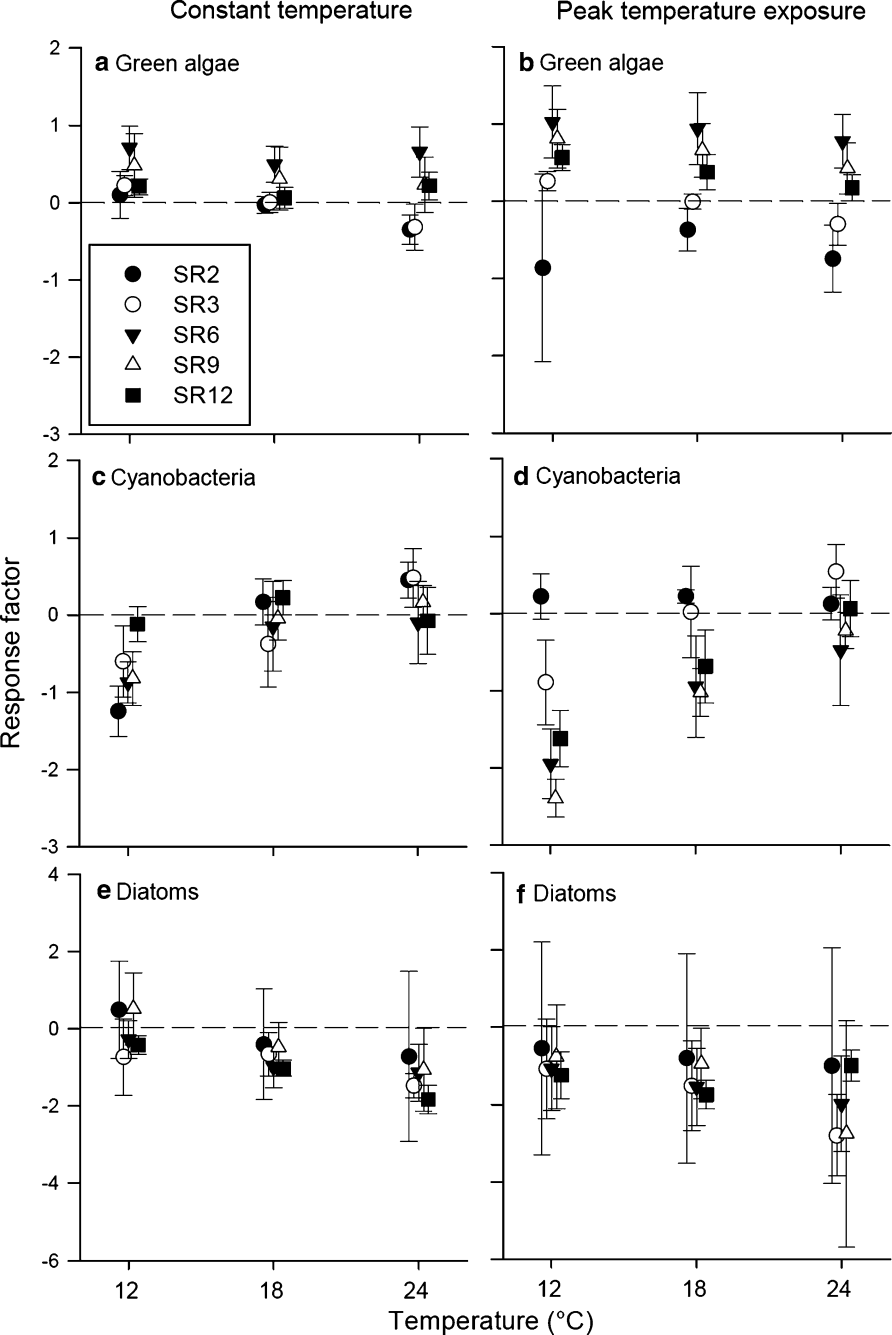
Algal response factors (mean ± SE) of mixed communities with various species richness (SR) levels (2, 3, 6, 9, or 12 species) as a function of constant (*t*_1_, *left column*) or peak (*t*_2_, *right column*) temperature exposure: **a**, **b** green algae, **c**, **d** cyanobacteria, **e**, **f** diatoms. The response factor is the relative biovolume development in mixed communities for each taxonomic group. Note different *y* *-axis* scaling for (**e**, **f**). Symbols are slightly displaced for better illustration

The response factor for green algae and diatoms tended to decrease with increasing temperature while the response factor of cyanobacteria tended to increase with increasing temperature (Table 1; Fig. 4). However, mean response factors for green algae were predominantly positive or not different to zero (Fig. 4), while cyanobacteria and diatoms showed mainly negative mean response factors or mean response factors not different from zero (Fig. 4).

**Table 1:** Linear regression analysis of algal response factor related to temperature

	Slope	Intercept	*r* ^2^	*P*
Green algae *t*_1_	−0.0202 (0.0148)	0.5797 (0.2753)	0.0272	0.1760
Green algae *t*_2_	−0.0462 (0.0195)	1.190 (0.3660)	0.0781	0.0210
Cyanobacteria *t*_1_	0.0685 (0.0206)	−1.4201 (0.3847)	0.1472	0.0015
Cyanobacteria *t*_2_	0.1283 (0.0269)	−3.0147 (0.5018)	0.2624	<0.0001
Diatoms *t*_1_	−0.1011 (0.0368)	1.1025 (0.6871)	0.1044	0.0082
Diatoms *t*_2_	−0.0242 (0.0555)	−0.7671 (1.0285)	0.0035	0.6640

For analysis, single data points were used while mean ± SE are displayed in Fig. 4

### Temperature effects on diversity

**Fig. 5:**
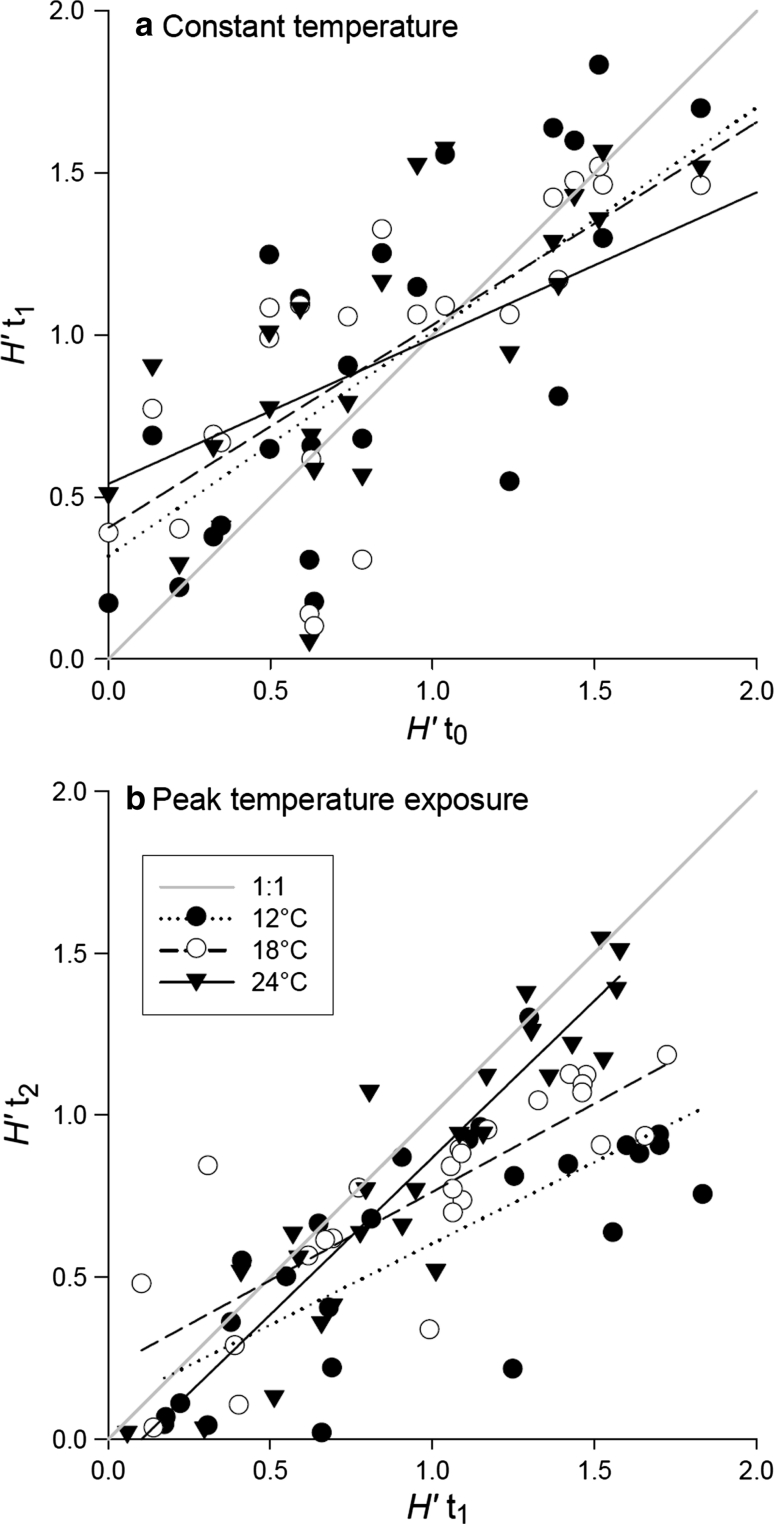
**a** Diversity after 2 weeks of constant temperatures (*H*′*t*_1_) as a function of initial diversity *(H*′*t*_0_) for the three experimental temperatures 12 °C (*H*′*t*_1_ = 0.28 + 0.67 × *H*′*t*_0_; *r* ^2^ = 0.57; *P* < 0.0001), 18 °C (*H*′*t*_1_ = 0.41 + 0.57 × *H*′t_0_; *r* ^2^ = 0.54; *P* < 0.001), and 24 °C (*H*′*t*_1_ = 0.52 + 0.44 × *H*′t_0_; *r* ^2^ = 0.39; *P* < 0.001) including linear regressions. **b** Diversity after temperature peaks (*H*′*t*_2_) as a function of diversity after 2 weeks of constant temperatures (*H*′*t*_1_) for the three experimental temperatures 12 °C (*H*′*t*_2_ = 0.1 + 0.5 × *H*′t_1_; *r* ^2^ = 0.55; *P* < 0.001), 18 °C (*H*′*t*_2_ = 0.22 + 0.54 × *H*′*t*_1_; *r* ^2^ = 0.67; *P* < 0.001), and 24 °C (*H*′*t*_2_ = −0.1 + 0.97 × *H*′*t*_1_; *r* ^2^ = 0.85; *P* < 0.001) including linear regressions. Both graphs include 1:1 line

While diversity (*H*′, Shannon Index) showed no significant differences between *t*_0_ and *t*_1_ at all temperatures (paired *t* test: 12 °C *P* = 0.52, 18 °C *P* = 0.74, 24 °C *P* = 0.56; Fig. 5a), diversity was generally lower after the short-term temperature peaks (*t*_2_) than after 2 weeks of constant temperature incubation (*t*_1_) at all three temperature levels (Fig. 5b; paired *t* test: 12 °C *P* < 0.001; 18 °C *P* < 0.001; 24 °C *P* = 0.001). The decrease of diversity from *t*_1_ to *t*_2_ was most pronounced at 12 °C.

## Discussion

### Positive species richness effects on growth rates and phosphorus content

The expected positive effect of species richness on growth rates and POP concentrations was confirmed at both constant temperature levels and after temperature peaks for all temperatures. The species richness-dependent increase in phytoplankton growth rates and POP concentrations was strongest when communities adapted to an intermediate temperature level (18 °C) experienced a short-term increase of temperature. It has been shown that light use efficiency increases with increasing species and functional diversity (Striebel et al. 2009b; Behl et al. 2011). Increased light use efficiency leads to enhanced C-fixation, yet does not necessarily promote P-uptake efficiency (Striebel et al. 2009a). In our study, both POC and POP increased significantly with increasing species richness until saturation was reached. This species richness-dependent increase of POP suggests that complementary processes other than light use efficiency influenced nutrient uptake in phytoplankton communities of higher species richness. However, a clear increase in C:P ratios after temperature peaks (Online Resource Fig. S4) suggests that the complementary effects of light use may be more pronounced compared to potential other complementary processes involved in nutrient uptake.

### Possible mechanisms of species richness effects on phytoplankton

Ptacnik et al. (2008) and Striebel et al. (2009a) showed that resource use and, in turn, biomass production are directly and positively linked to diversity in phytoplankton communities.

We found a clear effect of overyielding described as positive net effect at almost all temperature levels (except 12 °C at *t*_1_; Fig. 2). This overyielding could be explained by complementarity among species in the respective communities (Fig. 2), which was also positive whenever overyielding occurred. The selection effect was not significantly different from zero meaning that overyielding could not be explained by the selection effect (Fig. 2). Complementary traits or niche differentiation had been identified as the key mechanism behind the productivity enhancing effect of species and/or functional diversity in terrestrial plant communities (Loreau et al. 2001; Fargione et al. 2007; Flombaum and Sala 2008) and could also be confirmed for heterotrophic organisms in aquatic systems (Cardinale et al. 2002), as well as for phytoplankton (Striebel et al. 2009b). Complementarity leading to an enhancement of primary production in highly diverse phytoplankton communities can, at least partly, be explained by partitioning of the available photosynthetic active radiation through an increased taxa-specific variety of antenna pigment composition. However, potential mechanisms behind complementarity effects involving temperature have yet to be demonstrated.

### Temperature-dependent growth responses of different taxonomic groups

As expected, the 15 taxa of the three taxonomic groups examined showed temperature-dependent differences in growth rates (Online Resource Fig. S3). After pooling species in respect to their taxonomic group (green algae, cyanobacteria, and diatoms; Fig. 3), growth rates of each taxonomic group were similar and showed no effect of temperature, except for the growth rates of diatoms which were lower at 12 and 18 °C at *t*_1_. While at *t*_1_, diatom growth rates showed a trend to increase with temperature, at *t*_2_, all groups showed tendentially highest growth rates at 18 °C (Fig. 3).

When calculating the growth response (response factor; Fig. 4) of algae from different taxonomic groups grown in mixed cultures, results were slightly different. The mean response factor of green algae was highest at 12 °C and decreased with increasing temperature, while the mean response factor of cyanobacteria increased with increasing temperature (Table 1; Fig. 4). The response factor of diatoms tended to decrease with temperature (Table 1; Fig. 4) and had the highest variance within a taxonomic group (Fig. 4e, f). While the data gained from our monocultures do not necessarily support results in the existing literature, data from our mixed cultures are in line with the common view that in natural communities, diatoms prefer cooler waters, while cyanobacteria have high temperature optima for growth, and green algae generally perform better at intermediate temperatures (Canale and Vogel 1974; Paerl and Huisman 2008). These results show that species interactions, e.g., competitive behavior in communities of various phytoplankton taxa, constitute a key factor for the community development and species composition in phytoplankton communities.

### Negative effects of high temperatures on phytoplankton diversity

While we found no differences in diversity after 2 weeks of constant temperature treatments (Fig. 5a), diversity decreased at all temperature levels after short-term temperature peaks (Fig. 5b). This effect was strongest in those communities adapted to 12 °C, followed by the 18 °C treatments, and was less pronounced at 24 °C (Fig. 5b). This decrease in diversity after temperature peaks may have been enhanced by the longer total incubation time (one additional week after 2 weeks at constant temperatures). However, the differences between the temperature levels are unaffected by this experimental setup, which means that a temperature increase of 4 °C might have a less dramatic effect for algae already adapted to warmer temperatures. Looking at relative changes in temperature, an increase of 4 °C at 12 °C is equivalent to an increase of 33 %, while a temperature increase of 4 °C at 24 °C is equivalent to an increase of 16.6 %. Thus, the relative change in temperature might be more important for the diversity of phytoplankton communities than the absolute change in temperature.

## Conclusion

This study emphasized that biodiversity is an important factor determining phytoplankton community performance under varying temperature conditions. In general, higher diversity increases phytoplankton growth and phosphorus uptake, in our experiment especially at 18 °C. A temperature-dependent decrease in diversity was most obvious in communities adapted to cooler base temperatures, meaning that relative temperature changes might be more important than absolute changes for the diversity of phytoplankton communities. Green algae and diatoms showed a trend to perform better at lower temperatures, while cyanobacteria showed stronger responses with increasing temperatures in mixed communities. However, the data obtained from our monocultures did not support these trends, which indicates that species interactions in communities of various phytoplankton taxa are a major factor for the development and species composition in phytoplankton communities. Additionally, we found overyielding in almost all communities and complementarity as the underlying mechanism. Isolating single effects of altered temperature regimes in controlled laboratory experiments, and in artificial phytoplankton communities of rather low species richness, is an important contribution to the understanding of the principles behind these effects on aquatic ecosystems; in the next steps, however, the issue needs to be taken to the level of natural communities of greater diversity and more complex interaction potential.

## Electronic supplementary material

Below is the link to the electronic supplementary material.

Supplementary material 1 (DOCX 1574 kb)
