# Supplementary material for: Temperature and species richness effects in phytoplankton communities
Source: Oecologia. 2012 Aug 1;171(2):527–36. doi: 10.1007/s00442-012-2419-4 (PMC3548109; doi:10.1007/s00442-012-2419-4)
Supplement: Supplementary file 1 — Supplementary material 1 (DOCX 1574 kb) [file 442_2012_2419_MOESM1_ESM.docx]

**Online Resource.** “Temperature and species richness effects in phytoplankton communities”

Stefanie Schabhüttl, Peter Hingsamer, Gabriele Weigelhofer, Thomas Hein, Achim Weigert, and Maren Striebel

**Table 1** Phytoplankton species used in our experiments. Sources of algae cultures: ^1^Culture Collection of Algae Göttingen (SAG), Germany; ^2^Culture Collection of Autotrophic Organisms (CCLA), Třeboň, Czech Republic.

| **Species** | **Group** | **Order** | **Abbreviation** |
| --- | --- | --- | --- |
| *Chlamydomonas reinhardtii*^1^ | Chlorophyceae | Volvocales | Chl |
| *Pediastrum simplex*^1^ | Chlorophyceae | Chlorococcales | Ped |
| *Scenedesmus obliquus*^1^ | Chlorophyceae | Chlorococcales | Sce |
| *Spirogyra* sp.^1^ | Chlorophyceae | Zygnematales | Spi |
| *Staurastrum tetracerum*^1^ | Chlorophyceae | Desmidiales | Sta |
| *Anabaena cylindrica*^1^ | Cyanophyceae | Nostocales | Ana |
| *Aphanizomenon gracile*^1^ | Cyanophyceae | Nostocales | Aph |
| *Chroococcus minutus*^1^ | Cyanophyceae | Chroococcales | Chr |
| *Leptolyngbya fragilis*^2^ | Cyanophyceae | Oscillatoriales | Lep |
| *Oscillatoria limosa*^1^ | Cyanophyceae | Oscillatoriales | Osc |
| *Asterionella formosa*^1^ | Bacillariophyceae | Pennales | Ast |
| *Fragilaria crotonensis*^1^ | Bacillariophyceae | Pennales | Fra |
| *Navicula pelliculosa*^1^ | Bacillariophyceae | Pennales | Nav |
| *Skeletonema subsalsum*^1^ | Bacillariophyceae | Centrales | Ske |
| *Stephanodiscus minutulus*^1^ | Bacillariophyceae | Centrales | Ste |

**Table 2** 25 mixed communities of 5 species richness levels (2, 3, 6, 9, and 12). At each species richness level, five mixtures of different community composition were created by random selection of species from the 15 monocultures available. Mixture 5 was replicated three times for each species richness level. SR=species richness. Abbreviations of taxa names see Table 1.

| **SR** | **M** |  |  |  |  |  |  |  |  |  |  |  |  |
| --- | --- | --- | --- | --- | --- | --- | --- | --- | --- | --- | --- | --- | --- |
| 2 | 1 | Chl | Ped |  |  |  |  |  |  |  |  |  |  |
| 2 | 2 | Sce | Lep |  |  |  |  |  |  |  |  |  |  |
| 2 | 3 | Ped | Nav |  |  |  |  |  |  |  |  |  |  |
| 2 | 4 | Sce | Ana |  |  |  |  |  |  |  |  |  |  |
| 2 | 5 | Sta | Ske |  |  |  |  |  |  |  |  |  |  |
| 3 | 1 | Sce | Lep | Nav |  |  |  |  |  |  |  |  |  |
| 3 | 2 | Aph | Fra | Ske |  |  |  |  |  |  |  |  |  |
| 3 | 3 | Chl | Spi | Lep |  |  |  |  |  |  |  |  |  |
| 3 | 4 | Aph | Lep | Nav |  |  |  |  |  |  |  |  |  |
| 3 | 5 | Sce | Ped | Chr |  |  |  |  |  |  |  |  |  |
| 6 | 1 | Spi | Sce | Chr | Aph | Fra | Ske |  |  |  |  |  |  |
| 6 | 2 | Chl | Spi | Sce | Sta | Osc | Nav |  |  |  |  |  |  |
| 6 | 3 | Chl | Ped | Osc | Lep | Fra | Nav |  |  |  |  |  |  |
| 6 | 4 | Spi | Sce | Osc | Chr | Aph | Nav |  |  |  |  |  |  |
| 6 | 5 | Chl | Spi | Ana | Aph | Lep | Fra |  |  |  |  |  |  |
| 9 | 1 | Chl | Spi | Sce | Sta | Chr | Aph | Fra | Ast | Nav |  |  |  |
| 9 | 2 | Chl | Sce | Sta | Osc | Ana | Aph | Fra | Nav | Ste |  |  |  |
| 9 | 3 | Chl | Spi | Sce | Osc | Chr | Aph | Lep | Fra | Nav |  |  |  |
| 9 | 4 | Chl | Sce | Osc | Aph | Lep | Fra | Ast | Ste | Ske |  |  |  |
| 9 | 5 | Chl | Spi | Sce | Ana | Aph | Lep | Fra | Nav | Ske |  |  |  |
| 12 | 1 | Chl | Spi | Sce | Sta | Ped | Ana | Chr | Aph | Lep | Fra | Nav | Ske |
| 12 | 2 | Chl | Spi | Sce | Sta | Ped | Osc | Ana | Aph | Lep | Fra | Nav | Ske |
| 12 | 3 | Chl | Spi | Sce | Ped | Osc | Ana | Chr | Aph | Lep | Fra | Nav | Ske |
| 12 | 4 | Chl | Spi | Sce | Sta | Ped | Osc | Ana | Chr | Aph | Fra | Nav | Ske |
| 12 | 5 | Chl | Spi | Sce | Sta | Ped | Ana | Chr | Aph | Lep | Fra | Nav | Ske |

**Table 3** Variation coefficients (in %) for POC and POP of all mixtures per species richness level (including replicate 1 of mixture no. 5, excluding the remaining two replicates) and of the three replicates per mixture no. 5 (see Table 2) after two weeks of constant temperatures at t_1_ and after one additional week of short-term temperature peaks at t_2_ calculated as standard deviation divided by mean. SR=species richness.

|  |  | **t_1_** | | | | |  | **t_2_** | | | | |
| --- | --- | --- | --- | --- | --- | --- | --- | --- | --- | --- | --- | --- |
|  |  | **SR2** | **SR3** | **SR6** | **SR9** | **SR12** |  | **SR2** | **SR3** | **SR6** | **SR9** | **SR12** |
| **POC mixtures** | 12°C | 34.7 | 30.7 | 18.0 | 14.5 | 6.3 |  | 93.7 | 75.5 | 37.2 | 3.2 | 1.7 |
|  | 18°C | 36.8 | 48.9 | 17.6 | 12.8 | 6.9 |  | 39.5 | 51.5 | 28.2 | 9.7 | 5.5 |
|  | 24°C | 30.5 | 56.1 | 20.7 | 5.7 | 9.4 |  | 40.3 | 34.2 | 9.2 | 4.7 | 29.7 |
| **POC replicates** | 12°C | 2.2 | 2.0 | 2.0 | 3.0 | 4.3 |  | 6.2 | 3.9 | 5.0 | 4.8 | 6.9 |
|  | 18°C | 3.0 | 0.8 | 4.3 | 2.4 | 0.6 |  | 6.7 | 1.1 | 3.3 | 5.6 | 6.3 |
|  | 24°C | 3.9 | 1.0 | 4.2 | 32.1 | 3.7 |  | 9.3 | 3.9 | 5.5 | 4.0 | 8.8 |
| **POP mixtures** | 12°C | 14.8 | 14.2 | 36.0 | 11.6 | 31.7 |  | 50.5 | 33.0 | 29.2 | 8.1 | 3.8 |
|  | 18°C | 22.2 | 8.7 | 13.7 | 29.2 | 26.5 |  | 55.6 | 49.1 | 36.9 | 17.7 | 9.7 |
|  | 24°C | 6.7 | 15.7 | 51.8 | 36.6 | 18.5 |  | 39.6 | 51.2 | 34.1 | 18.9 | 31.2 |
| **POP replicates** | 12°C | 6.2 | 2.5 | 2.2 | 14.4 | 6.3 |  | 6.0 | 4.3 | 4.3 | 4.3 | 7.1 |
|  | 18°C | 1.1 | 4.7 | 10.2 | 5.1 | 5.4 |  | 12.0 | 6.5 | 14.3 | 6.6 | 13.5 |
|  | 24°C | 2.6 | 9.7 | 5.2 | 5.2 | 17.8 |  | 3.7 | 2.1 | 2.0 | 21.3 | 17.2 |

**Table 4** Variables of saturation curves (*y=a*x/(b+x)*; n=40) including standard errors for variables a and b for growth rates (r) and POP concentrations as a function of initial species richness after two weeks of constant temperatures (t_1_) and after short-term temperature peaks (t_2_) (Fig. 2). Significant *P*-values in bold.

|  |  | **t_1_** |  |  |  | **t_2_** |  |
| --- | --- | --- | --- | --- | --- | --- | --- |
|  | **12°C** | **18°C** | **24°C** |  | **12°C** | **18°C** | **24°C** |
|  |  |  |  |  |  |  |  |
| **r** |  |  |  |  |  |  |  |
| **a** | 0.13±0.01 | 0.18±0.01 | 0.17±0.01 |  | 0.19±0.01 | 0.19±0.01 | 0.18±0.01 |
| **b** | 0.06±0.15 | 0.33±0.15 | 0.26±0.15 |  | 0.60±0.18 | 0.42±0.12 | 0.42±0.15 |
| ***R^2^*** | 0.01 | 0.15 | 0.10 |  | 0.35 | 0.33 | 0.18 |
| ***P*** | 0.58 | **<0.05** | **<0.05** |  | **<0.001** | **<0.001** | **<0.001** |
|  |  |  |  |  |  |  |  |
|  |  |  |  |  |  |  |  |
| **POP** |  |  |  |  |  |  |  |
| **a** | 486.77±52.60 | 418.59±49.17 | 445.16±41.64 |  | 829.36±71.02 | 803.29±90.36 | 573.71±62.73 |
| **b** | 1.48±0.51 | 0.77±0.38 | 1.33±0.41 |  | 2.36±0.56 | 1.64±0.57 | 1.18±0.45 |
| ***R^2^*** | 0.42 | 0.23 | 0.46 |  | 0.69 | 0.43 | 0.34 |
| ***P*** | **<0.001** | **<0.01** | **<0.001** |  | **<0.001** | **<0.001** | **<0.001** |

**
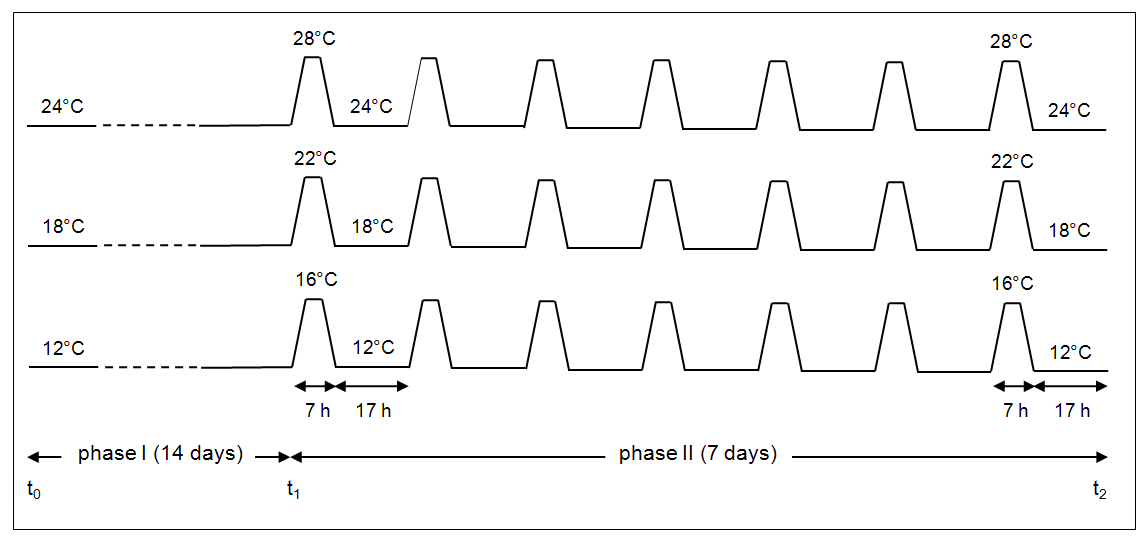
**

**Figure 1** Experimental setup: Phase I: 14-day incubation at constant temperatures (12°C, 18°C, 24°C). Phase II: 7-day incubation including daily 7-hour peaks of 4°C increase to reach 16°C, 22°C, and 28°C. Measure points at the start (t_0_), after phase I (t_1_), and after phase II (t_2_).

**Figure 2 (a-c)** Net biodiversity effect, **(d-f)** complementarity effect, and **(g-i)** selection effect as a function of species richness after two weeks of constant temperatures (a,d,g 12°C; b,e,h 18°C; c,f,i 24°C) at t_1_ (filled dots) and after one additional week of short-term temperature peaks at t_2_ (open dots) including significant linear regressions for t_2_ (no significant regressions at t_1_).


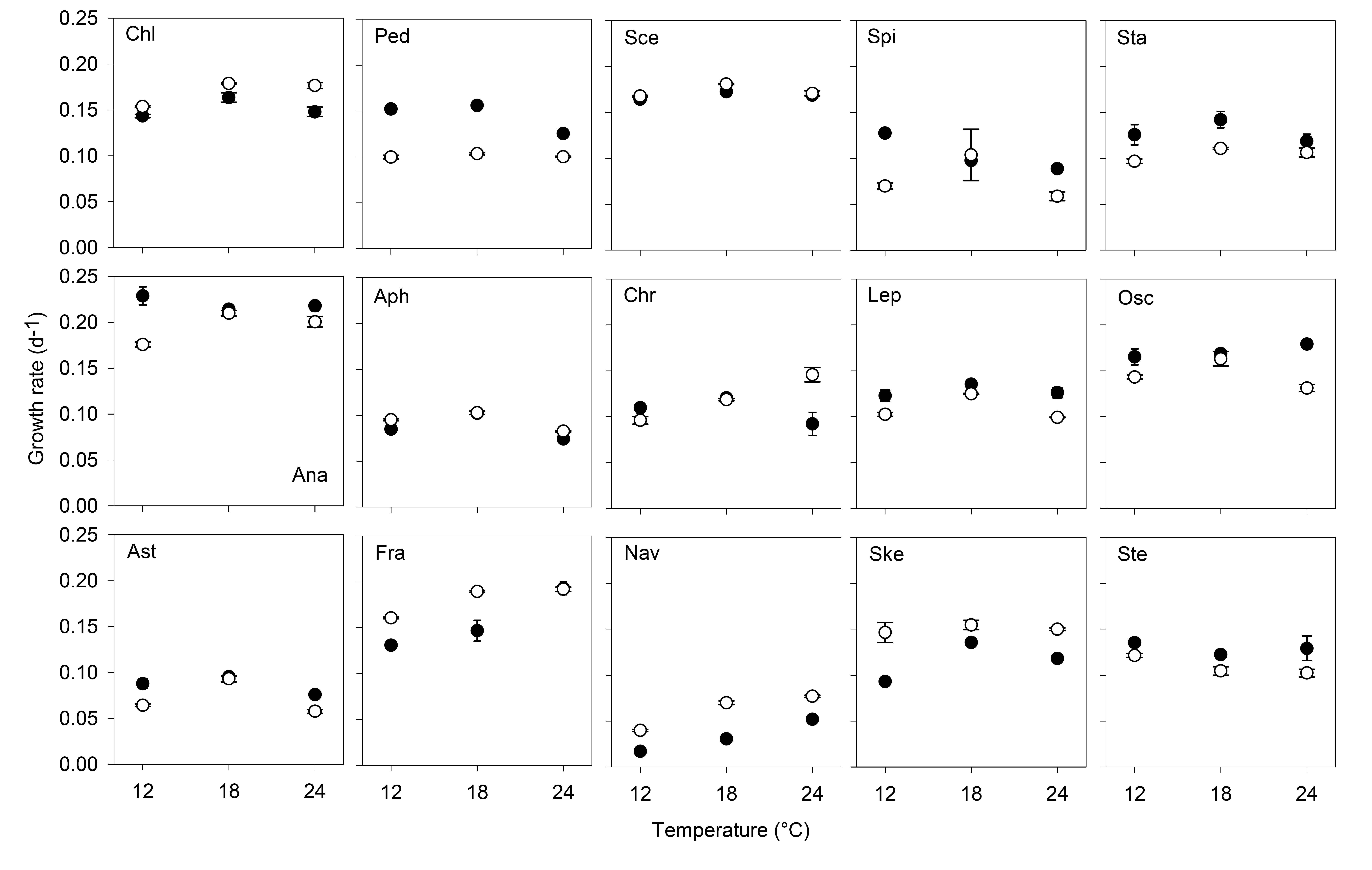
**Figure 3** Growth rates of monocultures (mean ± SE) as a function of temperature after two weeks of constant temperatures at t_1_ (filled dots) and after an additional week of short-term temperature peaks at t_2_ (open dots). Upper row green algae, middle row cyanobacteria, bottom row diatoms. Taxa name abbreviations see Online Resource Table 1.

**Figure 4** Molar C:P ratios as a function of initial species richness after two weeks of constant temperatures at t_1_ (a) and after one additional week of short-term temperature peaks at t_2_ (b) including significant regressions and saturation curves at t_2_ (12°C: C:P=45.6+2.71*SR, *r²*=0.2, *P*<0.01; 18°C: C:P=57.3+2.48*SR, *r²*=0.11, *P*<0.05; 24°C: C:P=(97.45*SR)/(0.74+SR), *r²*=0.19, *P*<0.01).
